# Effects of Applied Magnetic Field on the Optical Properties and Binding Energies Spherical GaAs Quantum Dot with Donor Impurity

**DOI:** 10.3390/nano12162741

**Published:** 2022-08-10

**Authors:** Collins Okon Edet, Emre Bahadir Al, Fatih Ungan, Norshamsuri Ali, Nursalasawati Rusli, Syed Alwee Aljunid, Rosdisham Endut, Muhammad Asjad

**Affiliations:** 1 Faculty of Applied and Human Sciences, Universiti Malaysia Perlis, Arau 02600, Malaysia; 2 Faculty of Electronic Engineering Technology, Universiti Malaysia Perlis, Arau 02600, Malaysia; 3 Department of Physics, Cross River University of Technology, Calabar 540252, Nigeria; 4 Physics Department, Faculty of Science, Cumhuriyet University, Sivas 58140, Turkey; 5 Institute of Engineering Mathematics, Universiti Malaysia Perlis, Arau 02600, Malaysia; 6 Department of Mathematics, Khalifa University, Abu Dhabi 127788, United Arab Emirates

**Keywords:** screened modified Kratzer potential (SMKP), refractive index, absorption coefficient, optical transitions, diagonalization method

## Abstract

The screened modified Kratzer potential (SMKP) model is utilized to scrutinize the impacts of an applied magnetic field (MF) on the binding energies and linear and nonlinear optical properties spherical GaAs quantum dot with donor impurity (DI). To accomplish this goal, we have used the diagonalization method to numerically solve the Schrödinger equation under the effective mass approximation for obtaining the electron energy levels and related electronic wave functions. The expressions used for evaluating linear, third-order nonlinear, and total optical absorption coefficients and relative refractive index changes were previously derived within the compact density matrix method. It has been shown here that the MF and DI impacts the characteristics of the absorption coefficients and the refractive index changes. This study’s results will find application in optoelectronics and related areas.

## 1. Introduction

Confinement potential (CP) models are analytical mathematical expresssions that represent the physical forces that acts on a particle within a specific region in space. It has been employed to model several physical phenomena. This is because it provides us with a relatively less-expensive approach to simulate physical systems of interest compared to experimetal and advanced computational approaches [1,2,3,4]. A number of physical phenomena have been modelled and simulated by several potential models [5,6,7,8,9], such as the quarkonium interactions have been modelled by Cornell potential [10,11,12], Yukawa potential [13,14,15], extended Cornell potential [16], etc. Diatomic and Polyatomic molecules have been modelled by the Morse potential [17,18,19,20,21], improved Tietz potential [22], Deng-Fan-Eckart potential [23,24,25] and many others. Plasma interactions have been modelled by Debye Huckel potential [26,27,28], Hulthen potential [29,30,31] and a host of others [32,33]. The applications of the potentials have also been witnessed in the emerging and ever advancing field of nanotechnology, as researchers have also modelled quantum dots (QDs) using Gaussian potential [34,35], modified Gaussian potential [35,36], Rosen-Morse potential [37] and many others. It has also been showed that the application of these models also aids in gathering some useful information like the thermodyanmic, transport, magnetic and optical properties of the above mentioned systems [3,22,38,39,40,41]. However, it will be necessary to point out that the application of the models mentioned above are not limited entirely to a specific area, based on appropriate manipulations and fittings of paramters, a model used to study diatomic molecules can be applied to study QD and vice versa. These possibilities do exist but there are exceptions particularly for cases concerning the diatomic molecules because the potential studying such a system must possess explcitly the dissociation energy, equilibrrium bond length, etc [42,43].

**Figure 1:**
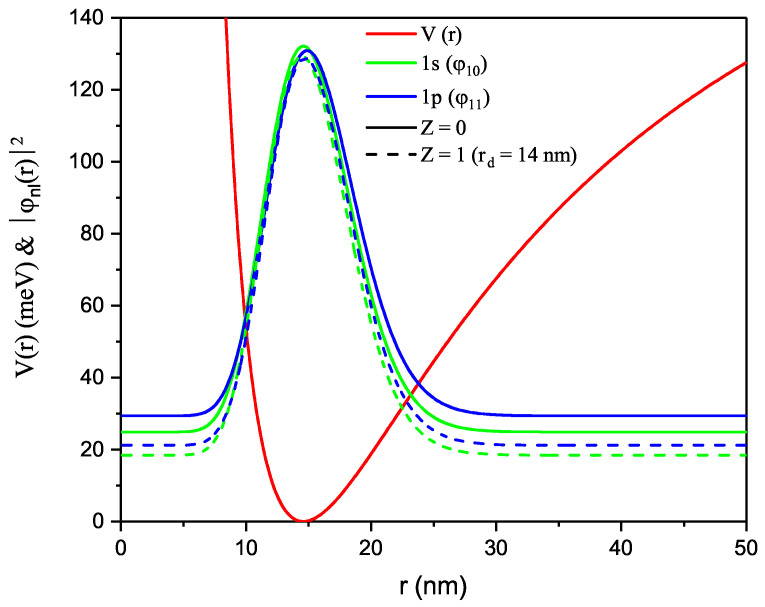
The confining SMKP (V(r)) and probability density $|\phi_{nl}{\left(r\right)|}^{2}$ as a function of *r*.

In this article, the SMKP [2,44] is adopted to model QDs. This SMKP has been applied by several authors to study diatomic molecules and other physical systems [2,44]. In order to broaden the scope of its applicability, this model is employed to simulate QDs since it poses the potential to study such systems (see Figure 1). Moreover, our choice is inspired by the fact that potential models with many fitting parameters tends to study physical systems and/or fit with experimental data better than those with fewer parameters and SMKP falls within this class (exponential-type potentials).

The study of the optical properties of QD has received immense attention from researchers in recent times. This is due to the exigent need for advanced nano-fabricated technologies. Optical properties of zero-dimensional structures (QD) has applications in the design process of electronic devices such as laser diodes, etc. [45,46]. It is on this basis that optical properties of semiconductor heterostructures have attracted unmatched consideration [46,47,48,49]. The study of optical properties of QD supplies us with such information like the optical absorption coefficients (OACs) [47], refractive index changes (RICs) [48,50], dipole transition [46,51], oscillator strength [52,53,54] and photoionization cross-sections [52]. Furthermore, this have enticed the interest of scholars in experimental and theoretical studies in recent years [46].

External fields such the MF, Aharanov-Bohm (AB) field, etc have been shown to modify and twist the optical properties of QDs in recent times. Notable amongst them is the removal degeneracy from the energy spectra by the magnetic and AB fields [2,4]. By modelling the QD with finite CP, Al et al [55] pointed out in their study that MF affects the impurity binding energies greatly when the dot radii is large also and the OACs. Again, Al et al. [46] have noted that the presence of MF removes degeneracy from the energies and shift the energy higher (blue shift). In addition, it has been showed that the cumulative effect of donor position, MF and dot size have been observed to make significant changes to the binding energies and OACs.

Having carefully highlighted the above, it is certain that the relevant motive for the present study is established. Being relevant to the field of device applications, the QDs are systems that exhibit remarkable optical and electronic properties. These can be properly tuned by suitably modifying the composition, structural parameters, and the application of external probes. Here, the optical properties and binding energies of donor impurity of a spherical GaAs QD structure with SMKP confinement potential have been investigated under the influence of externally applied MF, as well as the change of structure parameters. This is done with the purpose of analyzing the effects on the energy spectrum of externally applied fields as well as structural modifications. From such an outcome, the coefficients of linear and nonlinear optical absorption and refractive index change will be separately analyzed. The organization of the article is as follows: in Section 2, the formulation and theoretical solution of the problem are given, numerical results and their discussions are presented in Section 3 and our conclusions are given in Section 4.

## 2. Theoretical Framework

For an electron in motion in a spherical QD with the nonexistence of DI in the effective mass (EM) approximation, the Hamiltonian is given as [55];

(1) $$H^{\left(0\right)}=\frac{{\vec{p}}^{2}}{2m^{*}}+V\left(r\right)$$

where $\vec{p}$ is the momentum operator $m^{*}$ is the electron EM, and $V\left(r\right)=D_{e}{\left(q-r_{e}e^{-\alpha r}/r\right)}^{2}$ is the spherically symmetric SMKP confinement potential with $D_{e}$ is the dissociation energy, $r_{e}$ is the internuclear distance, α is the screening parameter, *q* is the control parameter and *r* is the internuclear distance [2,44].

The Hamiltonian of the system comprising of the MF and donor impurity (DI) atom located at the center of the finite spherical QD is given by [55]:

(2) $$H=\frac{1}{2m^{*}}{\left(\vec{p}+\frac{e}{c}\vec{A}\right)}^{2}+V\left(r\right)-\frac{e^{2}Z}{\varepsilon |\vec{r}|},$$

where *e*, $\vec{A}$, ε, $\vec{r}$ and *c* are charge of the electron, vector potential (VP) of the MF, the stationary dielectric constant of the QD material, electron position vector and the speed of light in vacuum, respectively with Z=0(Z=1) accounts for absence (presence) of impurity. This VP $\vec{A}$ is selected such that the MF is $\vec{B}=\vec{▿}\times \vec{A}$. Invoking the Coulomb gauge ($\vec{▿}.\vec{A}=0$), the VP satisfies the relationship $\vec{A}=\vec{B}\times \vec{r}/2$. Considering that the MF is applied along the z-axis, the Hamiltonian (Equation (2)) in its dimensionless expression is given as [46]

(3) $$H=\frac{1}{r^{2}}\frac{\partial }{\partial r}\left(r^{2}\frac{\partial }{\partial r}\right)+\frac{l(l+1)}{r^{2}}+V\left(r\right)+m\gamma +\frac{\gamma^{2}r^{2}\sin^{2}\left(\theta \right)}{4}-\frac{2Z}{r},$$

where $\gamma =ea_{B}^{2}\vec{B}/\hbar c$ with $a_{B}=\hbar^{2}\varepsilon /m^{*}e^{2}$ is the effective Bohr radius. Furthermore, *l* and *m* are the angular and magnetic momentum quantum numbers, correspondingly. The energy levels in the presence (Z=1) and absence (Z=0) of DI and their associated wave functions are derived by solving the eigenvalue corresponding to the Hamiltonian in Equation (3) is given as [53];

(4) $$H\psi_{nlm}\left(r,\theta ,\phi \right)=E_{nlm}\psi_{nlm}\left(r,\theta ,\phi \right),$$

where *n* is the principal quantum number and ϕ is the angle between the $\vec{r}$-projection on the xy-plane and the x-axis. Under the influence of MF and in the presence of DI atom in the QD system, Equation (4) is not solveable analytically. The solutions of Equation (4) can be obtained by employing the approach that is founded on the expansion of the electronic states over a complete orthogonal basis as presented as follows [55,56]

(5) $$\psi_{nlm}\left(r,\theta ,\phi \right)=\sum_{j=1}^{\sigma }C_{nlm}\psi_{n_{j}l_{j}m}^{\left(0\right)}\left(r,\theta ,\phi \right),$$

where σ is the size of the basis set with an accurateness of 0.001 meV of the energy value. $C_{n_{j}l_{j}}$ is the *j*-th coefficient of the expansion (where the electronic states are categorized matching to a particular *m* value) and $\psi_{n_{j}l_{j}m}^{\left(0\right)}\left(r,\theta ,\phi \right)$ are the exact solutions of the infinite QD (where the radius is three times that of the QD with finite confinement potential V(r)). We point out here that when the total Hamiltonian matrix elements are computed, The wave function expansion will incorporate the terms of the finite confinement potential $V_{0}\equiv D_{e}$ that exists in the interval a<r<3a. The MF is known to eliminate the spherical symmetry of the problem, the orbital quantum number is an invalid quantum number, nonetheless three quantum numbers (n,l,m) are utilized to illustrate the new states suitably. In the absence of MF and DI [55]

(6) $$\psi_{n_{j}l_{j}m}^{\left(0\right)}\left(r,\theta ,\phi \right)=R_{nl}^{\left(0\right)}\left(r\right)Y_{lm}\left(\theta ,\phi \right),$$

where $R_{nl}^{\left(0\right)}\left(r\right)$ is the radial part of the non-correlated electron eigenfunction. $Y_{lm}\left(\theta ,\phi \right)=e^{im\phi }P_{lm}\left(\cos\left(\theta \right)\right)$ with $P_{lm}\left(\cos\left(\theta \right)\right)$ being the associated Legendre polynomial. Notice that *m* is an integer number which guarantee the azimuthal symmetry with respect to the axial z-axis. The impurity binding energy is given by $E_{nlm}^{b}=E_{nlm}\left(Z=0\right)-E_{nlm}\left(Z=1\right)$, where $E_{nlm}\left(Z=0\right)$ and $E_{nlm}\left(Z=1\right)$ are the energies of the electron in the absence and presence of DI, respectively. Intersubband transitions involves the ground and the first excited subband, once the incident photon energy ℏω is equal to the difference in energy between subbands, this is mathematically given as; $\hbar \omega =E_{21}=E_{2}-E_{1}$, where $E_{1}$ and $E_{2}$ are the ground and first excited states energy levels. In this study, we used z direction and x-y plane polarized EM radiation. A medium polarization is stimulated by the subband transition as a result of the ordering of the electric dipole moments in the direction of EM field [35,37,57]. The mathematical expressions of the linear $\chi^{\left(1\right)}$ and the third-order nonlinear $\chi^{\left(3\right)}$, using the density matrix approximation, susceptibility coefficients are presented as follows

(7) $$\begin{array}{ccc} \varepsilon_{0}\chi^{\left(1\right)} & = & \frac{\sigma_{\nu }{\left|M_{21}\right|}^{2}}{E_{21}-\hbar \omega -i\hbar \Gamma_{12}}, \end{array}$$

(8) $$\begin{array}{ccc} \varepsilon_{0}\chi^{\left(3\right)} & = & \frac{\sigma_{\nu }{\left|M_{21}\bar{E}\right|}^{2}}{E_{21}-\hbar \omega -i\hbar \Gamma_{12}} [\frac{4|M_{21}{|}^{2}}{{\left(E_{21}-\hbar \omega \right)}^{2}+{\left(\hbar \Gamma_{12}\right)}^{2}}-\frac{4|M_{22}-M_{21}{|}^{2}}{{\left(E_{21}-i\hbar \Gamma_{12}\right)}^{2}+{\left(E_{21}-\hbar \omega -i\hbar \Gamma_{12}\right)}^{2}}], \end{array}$$

where $M_{ij}$ is the matrix elements of the electric dipole moment, $E_{ij}=E_{i}-E_{j}$ is the energy difference between the two electronic states, ℏω is the incident photon energy, $\sigma_{v}$ is the carrier density and $\Gamma_{12}$ is the relaxation rate. The matrix elements of electric dipole moment can be evaluated using $M_{ij}=\langle \psi_{j}\left|er\sin\left(\theta e^{\pm i\phi }\right)\right|\psi_{j}\rangle$ for i,j=1,2. Therefore, the dipole transition matrix element in general is written as

(9) $$M_{ij}^{\left(xyz\right)}=\left\{\begin{array}{cc} M_{ij}^{\left(z\right)} & if\Delta m=0\\ M_{ij}^{\left(xy\right)} & if\Delta m=\pm 1. \end{array}\right.$$

The intersubband optical transition probability amplitude between the two electronic states, such as the lowest (initial) and the first excited (final), is proportional to the matrix element of electric dipole moment. Because the electric dipole moment is unaffected by spin, optical transitions can only occur between spin states with the same spin. When the selection rules Δl=±1 and Δm=0,±1 satisfied, the matrix elements of electric dipole moment are non-vanishing [46,48]. The OAC-α(ω) is obtained from the susceptibility χ(ω) using the relation given as $\alpha \left(\omega \right)=\omega \sqrt{\mu_{0}/\varepsilon }ℑ\left[\varepsilon_{0}\chi \left(\omega \right)\right]$, where $\mu_{0}$ vacuum permeability and $\varepsilon =n_{r}^{2}\varepsilon_{0}$ permittivity of the material with $n_{r}$ is the refractive index. The linear and third-order nonlinear OACs can be expressed as

(10) $$\begin{array}{ccc} \alpha^{\left(1\right)}\left(\omega \right) & = & \omega \sqrt{\mu_{0}/\epsilon }\frac{\hbar \sigma_{v}\Gamma_{12}{\left|M_{21}\right|}^{2}}{{\left(E_{21}-\hbar \omega \right)}^{2}+\hbar^{2}\Gamma_{12}^{2}},\\ \alpha^{\left(3\right)}\left(\omega ,I\right) & = & \frac{-\hbar I\omega \sqrt{\mu_{0}/\epsilon }\sigma_{v}\Gamma_{12}{\left|M_{21}\right|}^{2}}{2\varepsilon_{0}n_{r}c{\left[{\left(E_{21}-\hbar \omega \right)}^{2}-\hbar^{2}\Gamma_{12}^{2}\right]}^{2}}\left\{4|M_{21}{|}^{2}\right. \end{array}$$

(11) $$\begin{array}{ccc} & - & \left.\frac{|M_{22}-M_{11}{|}^{2}\left[3E_{21}^{2}-4\hbar \omega E_{21}+\hbar^{2}\left(\omega^{2}-\Gamma_{12}\right)\right]}{E_{21}^{2}+\hbar^{2}\Gamma_{12}^{2}}\right\}, \end{array}$$

where $I=2\varepsilon_{0}n_{r}c{\left|\bar{E}\right|}^{2}$ represents the incident optical intensity. Because of the spherical symmetry of the system ($M_{ii}=0$), the third-order nonlinear OAC-$\alpha^{\left(3\right)}\left(\omega ,I\right)$ is negative and also it is proportional to the incident optical intensity-*I*. Then, the total OAC α(ω,I) is given by $\alpha \left(\omega ,I\right)=\alpha^{\left(1\right)}\left(\omega ,I\right)+\alpha^{\left(3\right)}\left(\omega ,I\right)$. Since $|M_{jj}-M_{ii}|$ equals to zero for the on-center donor impurity, the third order nonlinear OAC becomes $\alpha^{\left(3\right)}\left(\omega ,I\right)=-\sqrt{\mu_{0}/\varepsilon_{r}}2\hbar \omega I\sigma_{v}\Gamma_{12}{\left|M_{21}\right|}^{4}/\varepsilon_{0}n_{r}c\left[{\left(E_{21}-\hbar \omega \right)}^{2}+\hbar^{2}\Gamma_{12}^{2}\right]$. Since the value of the $\hbar \Gamma_{ij}$ can not be ignored and the corresponding resonance condition is given by $\hbar \omega \sqrt{E_{ij}^{2}+\hbar^{2}\Gamma_{ij}^{2}}$. The RIC Δn(ω) can be obtained from susceptibility χ(ω) by $\Delta n\left(\omega \right)/n_{r}=ℜ\left[\chi \left(\omega \right)/2n_{r}^{2}\right]$ [38,46]. Then the linear $\Delta n^{\left(1\right)}\left(\omega \right)$ and third-order nonlinear $\Delta n^{\left(3\right)}\left(\omega \right)$ RICs are given by

(12) $$\begin{array}{ccc} \frac{\Delta n^{\left(1\right)}\left(\omega \right)}{n_{r}} & = & \frac{\sigma_{v}{\left|M_{21}\right|}^{2}}{2n_{r}^{2}\varepsilon_{0}}\frac{E_{21}-\hbar \omega }{{\left(E_{21}-\hbar \omega \right)}^{2}+\hbar^{2}\Gamma_{12}},\\ \frac{\Delta n^{\left(3\right)}\left(\omega \right)}{n_{r}} & = & \frac{\sigma_{v}{\left|M_{21}\right|}^{2}\mu_{0}cI}{4n_{r}^{2}\varepsilon_{0}}\left\{\frac{4\left(E_{21-\hbar \omega }\right){\left|M_{21}\right|}^{2}}{{\left(E_{21}-\hbar \omega \right)}^{2}-\hbar^{2}\Gamma_{12}^{2}}\right. \end{array}$$

(13) $$\begin{array}{ccc} & - & \left.\frac{|M_{22}-M_{11}{|}^{2}E_{21}-\hbar \omega \left(E_{21}{\left(E_{21}-\hbar \omega \right)}^{2}-\hbar^{2}\Gamma_{12}^{2}\right)-\hbar^{2}\Gamma_{12}^{2}\left(2E_{21-\hbar \omega }\right)}{\left[{\left(E_{21}-\hbar \omega \right)}^{2}-\hbar^{2}\Gamma_{12}^{2}\right]\left[{\left(E_{21}-\hbar \omega \right)}^{2}+\hbar^{2}\Gamma_{12}^{2}\right]}\right\}. \end{array}$$

It is clear from Equations (12) and (13), the third-order nonlinear RIC-$\Delta n^{\left(3\right)}/n_{r}$ has contrasting sign with the sign of $\Delta n^{\left(1\right)}/n_{r}$ and it is proportionate to the incident optical intensity-*I*. Therefore, the OACs (Eqautions (10) and (11)) and RICs (Eqautions (12) and (13)), depends only on the matrix element $M_{21}$ of the dipole moment. Once a photon with energy-ℏω is released into the system, the matrix element $M_{21}$ provides the probability amplitude of one electron optical transition between the state |1〉 and |2〉 described by the wave functions.

## 3. Results and Discussion

This study presents the optical properties of the GaAs QD structure using SMKP. Numerical computations are carried out for this QD structure. Fitting parameters used to achieve our set objectives are presented as follows: μ=0.067 me, $\sigma_{v}=5\times 10^{22}$ m${}^{-3}$ and $n_{r}=3.2$ [35]. Consequently, $\varepsilon_{0}=8.854\times 10^{-12}$ F/m is the vacuum permittivity, $\mu_{0}=4\pi^{-7}$ H/m is the vacuum permeability, and $\Gamma_{12}=1/T_{12}$ where $T_{12}=0.14$ ps, $D_{e}=224.46$ meV, $r_{e}=1.5$, q=1, α=0.05, $m_{e}=0.067\;m_{0}$, ε=13.18, $\sigma =5\times 10^{22}$ m${}^{-3}$, I=200 MW/m${}^{2}$ and $a_{B}=104.24$ A.

The experimental value for the depth of the potential well-$D_{e}$ and radius of the dot- $R_{0}$ are selected in line with what obtains in literature [57]. It is important to point out here that $\sigma^{+}$ corresponds to transition between $1p_{+1}$ and 1s state ($1p_{+1}\to 1s$), $\sigma^{-}$ corresponds to transition between $1p_{-1}$ and 1s ($1p_{-1}\to 1s$) and π transition between $1p_{0}$ and 1s ($1p_{0}\to 1s$). In addition, *p* corresponds to l=1, while *s* corresponds to l=0. *l* is the angular momentum quantum number, −1, 0 and +1 are *m* values and *m* is the magnetic momentum quantum number.

The SMKP is the adopted confining potential for this study, it has finite depth and range. This potential is a continuous function that has four parameters that can be adjustable at will to fit a physical system of interest. In this study, our goal is to study the optical properties of a QD with the SMKP with DI in the presence of an applied MF. Historically, it has been established that for a potential model to be used to study intersubband transition occurrences and related properties of QDs, the potential must be of a quadratic-like form just like the SMKP (see Figure 1). Figure 1 shows the confining SMKP as a function of *r*. The shape of this model is in agreement with the shape of other confinement potential adopted previously for such considerations [36,37,58]. However, our model is an improvement to the aforementioned in the sense that our potential has more fitting parameters and it is a general case of the Kratzer potential [59]. Figure 1 also shows the probability density, ${\left|\phi \left(r\right)\right|}^{2}$ for 1s and 1p states and in the presence and absence of DI. It is seen that probability density increases for higher states but there is no discernable difference (overlap) in the peak positions and this implies that the particle has an intense localization. Probability density offers information on the concentration of the density distribution.

**Figure 2:**
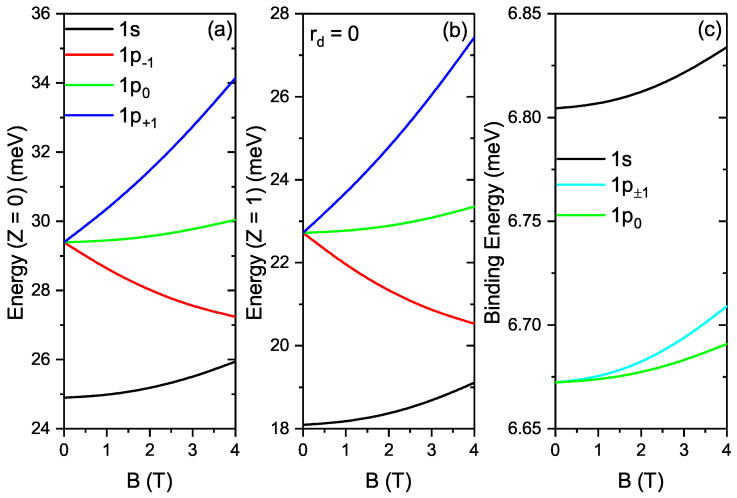
(**a**) Electron energies versus the MF in the absence of impurity; (**b**) Electron energies versus the magnetic field in the presence of DI. (**c**) Binding energies versus the MF.

Figure 2a,b show the electron energies versus the MF in the absence and presence of DI for 1s, $1p_{-1}$, $1p_{0}$ and $1p_{+1}$. However, for $1p_{-1}$, $1p_{0}$ and $1p_{+1}$ states, there is a convergence at B=0T and with MF there is a splitting which definitely implies elimination of degeneracy due to Zeeman effect. Figure 2c shows the binding energies versus the MF for 1s, $1p_{\pm 1}$ and $1p_{0}$. The energy increases in the order presented as follows; $1p_{0}$, $1p_{\pm 1}$ and 1s. The binding energy is also seen to rise with rising MF.

**Figure 3:**
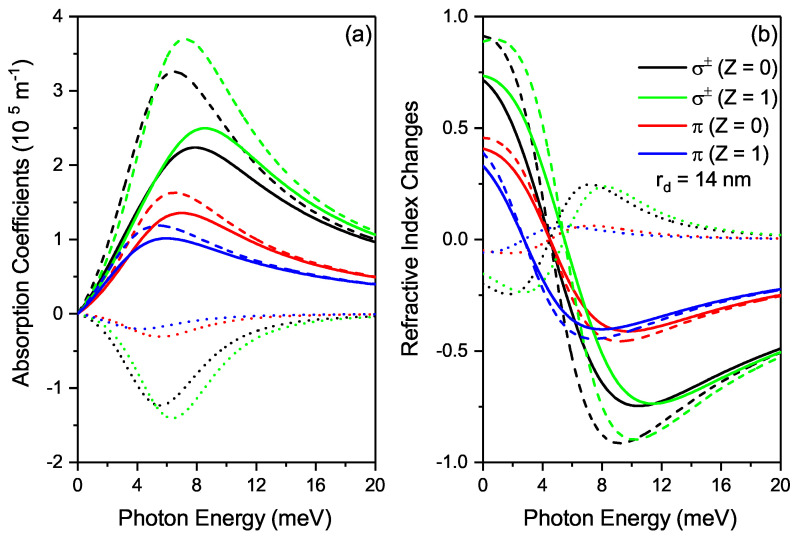
(**a**) The plot of optical absorption coefficient as a function of the photon energy. (**b**) The Refractive index changes as a function of the photon energy. Both plots are for $\sigma^{\pm }$(Z=0), $\sigma^{\pm }$(Z=1), π(Z=0) and π(Z=1) transitions. In (**a**,**b**), the dashed, doted and solid curves are for linear, third-order nonlinear and total optical absorption coefficients (Refractive Index Changes) respectively.

Figure 3a shows the OACs as a function of the photon energy for $\sigma^{\pm }$(Z=0), $\sigma^{\pm }$(Z=1), π(Z=0) and π(Z=1) transitions. To study the OACs of this system, the DI position is fixed at $r_{d}=14$ nm. It is observed that the linear and total OACs first increase and then decrease as the photon energy increases while negative nonlinear OAC shows the contrary variation pattern, this observed in both transitions. A quasi-resonance peak is observed about photon energy of ≈8 meV for total OAC. The OAC is higher in the presence of DI than in the absence for $\sigma^{\pm }$-transition, while it is lower for π-transition. The DI shifts the peak position of linear and nonlinear OACs slightly towards blue (high energy) due to the increase in transition energy, while the absence of the DI causes these peaks to shift to red (low energy) in the $\sigma^{\pm }$-transition and the converse is observed in π-transition. We may conclude here that the influence of these DI in the case of the $\sigma^{\pm }$ and π-transitions on the OACs is great and can be instrumental in the manipulation of the OACs. In Figure 3b we plot RICs as a function of the photon energy for $\sigma^{\pm }$(Z=0), $\sigma^{\pm }$(Z=1), π(Z=0) and π(Z=1) transitions. The RICs firstly decrease and then slightly increases with rising of photon energy as shown in Figure 3b. When the DI is present, the RICs increase (decrease) for $\sigma^{\pm }$-(π-)-transition and their positions move to the greater (smaller) energies. This is attributed to the fact that the energy difference between the two electronic states increases for $\sigma^{\pm }$-transition, while it decreases for π-transition.

**Figure 4:**
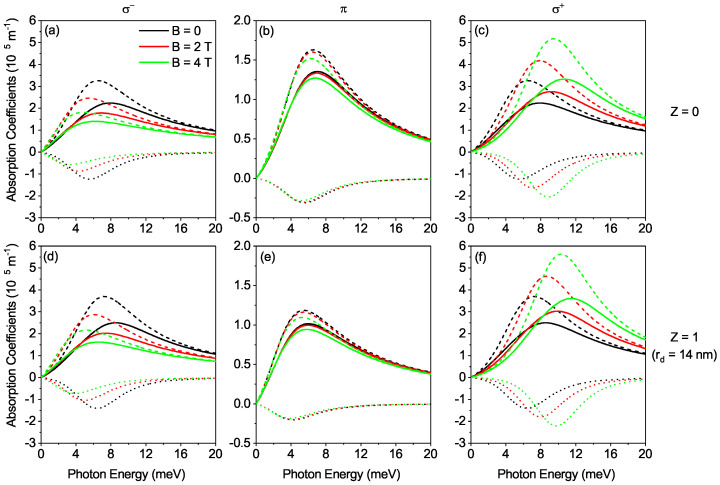
Plot of optical absorption coefficients as a function of the photon energy for $\sigma^{\pm }$(Z=0), $\sigma^{\pm }$(Z=1), π(Z=0) and π(Z=1) transitions and varying MF (**a**–**f**). The dashed, doted and solid curves represent linear, third-order nonlinear and total optical absorption coefficients respectively.

Figure 4a,d show the OACs as a function of the photon energy for $\sigma^{-}$-transition in the present presence and absence of DI respectively with varying MF. The linear and total OACs rise first and decrease with rising photon energy, but their resonant peaks decrease with rising MF. However, there is no discernable difference in the OACs for all values of *B*-field between the presence and absence of DI for the considered transition. In Figure 4b,e, the linear and total OACs again rises first and decrease with rising photon energy, but they do not show a discernable variation pattern as the MF rises for the transition in the presence and absence of DI In addition, the presence of DI reduces the peak amplitude of the π-transition while red-shifting the peak position. Figure 4c,f shows the variation of the OACs as a function of the photon energy with varying MF in the absence and presence of DI for $\sigma^{+}$-transition. The linear and total OACs first increase and then decrease as the photon energy increases while negative nonlinear OAC shows the opposite change. The increase in *B*-field parameters shifts the peak position of OACs slightly towards blue (high energy) due to the increase in transition energy. In addition, the peak amplitudes of linear and nonlinear OACs increase with the increase of B-field, due to more overlapping of the wavefunctions of the ground and first excited states and hence the increase in the dipole matrix element. Moreover, the peak amplitude of the total OAC also increase with the increase of the MF, since the positive increase in the peak amplitude of the linear OAC is dominant on the negative increase in the peak amplitude of the nonlinear OAC. However, there is no discernable difference in the OAC for the absence and presence of DI. We may conclude here that the influence of the effects of MF in $\sigma^{\pm }$-transitions and the effects of DI in π-transitions are abundant and these effects play an influential role in the control of the behavior of OACs.

**Figure 5:**
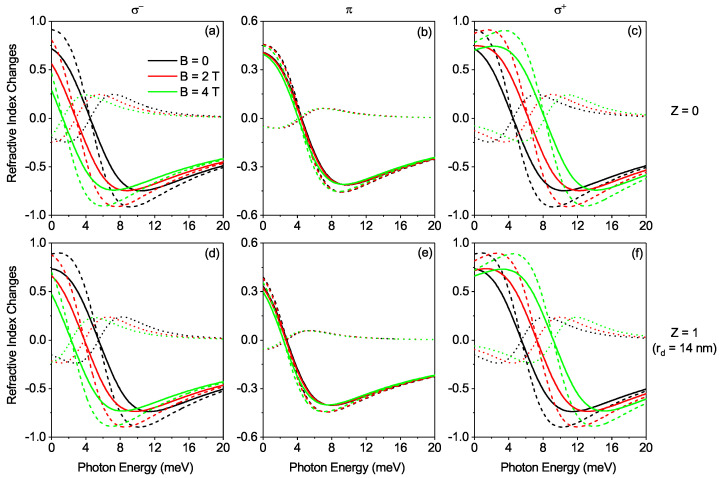
Plot of refractive index changes as a function of the photon energy for fixed values of $\sigma^{\pm }$(Z=0), $\sigma^{\pm }$(Z=1), π(Z=0) and π(Z=1) transitions and varying MF (**a**–**f**). The dashed, doted and solid curves account for linear, third-order nonlinear and total refractive index changes respectively.

Figure 5 shows the RICs as a function of the photon energy for $\sigma^{\pm }$(Z=0), $\sigma^{\pm }$(Z=1), π(Z=0) and π(Z=1) transitions. In Figure 5a,c,d,f, the RICs firstly decrease and then increase with increasing photon energy but slightly shifts upward as a function of the photon energy for $\sigma^{+}$-transitions when the MF is rising and but the contrary is observed in the $\sigma^{-}$-transitions. This is attributed to the fact that the energy difference between the two electronic states changes as shown in Table 1. In Figure 3b,e shows the same behavior with rising of photon energy for total RIC in the π-transition in the presence and absence of DI. There is again no discernable difference in the trend for π-transition as the MF rises. Again, we note here that the impact of the effects of MF in $\sigma^{\pm }$-transitions and the effects of DI in π-transitions are ample and these effects play a significant function in the regulation of the behavior of OACs.

## 4. Conclusions

This study presents the effects of applied MF on the optical properties and binding energies (for $\sigma^{\pm }$ and π) of donor DI in spherical GaAs quantum dot. In view of this, the SMKP potential has been utilized. To derive the eigenvalues equations and wave functions, the Schrodinger equation in spherical coordinate was solved by diagonalization approach. On variation of the *B*-field in the presence and absence of DI, the absorption coefficients and refractive index changes switch from lower to higher energies (blue shift) and also from higher to lower energies (red shift) for several transitions considered. It can be inferred that the MF and DI are greatly instrumental for manipulation of the optical properties of the GaAs quantum dot. Our results are vital in the design of nano-scale electronic devices such as quantum dot solar cells, quantum dot lasers, quantum dot photodetectors, quantum dot infrared photodetectors, phototransistors, and light-emitting diodes.

## Figures and Tables

**Table 1:** The subband energy differences and dipole moment matrix elements for (a), (b), (c), (d), (e) and (f) respectively, in Figure 4 and Figure 5.

B	0	2T	4T
$E_{ij}$	4.49174	2.83055	1.29578
$M_{ij}$	13.9468	13.9282	13.8737
$E_{ij}$	4.49174	4.39326	4.10013
$M_{ij}$	9.86191	9.85168	9.82146
$E_{ij}$	4.49174	6.28157	8.19782
$M_{ij}$	13.9468	13.9282	13.8737
$E_{ij}$	5.4793	3.81847	2.28483
$M_{ij}$	13.824	13.8066	13.7554
$E_{ij}$	2.75256	2.65404	2.36068
$M_{ij}$	9.74208	9.73287	9.70561
$E_{ij}$	5.4793	7.26949	9.18687
$M_{ij}$	13.824	13.8066	13.7554

## Data Availability

The datasets used and/or analyzed during the current study are available from the corresponding author on reasonable request.
